# Laparoscopic approach to an incidentally found pelvic retroperitoneal liposarcoma: Case report and review

**DOI:** 10.1097/MD.0000000000015184

**Published:** 2019-04-12

**Authors:** Vincenzo Dario Mandato, Valentina Mastrofilippo, Loredana De Marco, Lorenzo Aguzzoli

**Affiliations:** a Unit of Obstetrics and Gynecology; b Unit of Surgical Gynecol Oncology; c Unit of Pathology, Azienda USL-IRCCS of Reggio Emilia, Risorgimento, Italy.

**Keywords:** disease-free survival, laparosocopy, ovarian teratoma, radical resection, retroperitoneal liposarcoma

## Abstract

**Rationale:**

Well-differentiated liposarcomas (WDLPS) are rare retroperitoneal tumors that can reach significant size as they can grow without constrains before becoming symptomatic. Laparotomic open radical tumor resection represents the most common surgical approach.

**Patient concerns:**

A mass with “fat fluid level” was found in the right pelvis of an asymptomatic woman undergoing routine transvaginal ultrasound: the preoperative diagnosis was right mature ovarian teratoma.

**Diagnosis:**

Postoperative histopathology confirmed the diagnosis of WDLPS.

**Interventions:**

A radical laparoscopic excision of the retroperitoneal mass with bilateral salpingectomy was performed.

**Outcomes:**

Patient is free of disease at 18 months after surgery.

**Lesson:**

Despite computed tomography scan is the gold standard technique to identify WDLPS, such neoplasms can be misdiagnosed for mature ovarian teratomas. When a retroperitoneal mass is incidentally discovered during a surgery, an open core-needle biopsy is usually performed, and appropriate treatment planned only after complete staging and final pathology are available. Instead, when tumor margins are identified, resection of an incidentally diagnosed WDLPS would benefit from laparoscopic magnification that could improve distinguishing the disease from the surrounding tissues. Therefore, laparoscopy could represent a safe and effective technique to diagnose and treat retroperitoneal diseases.

## 1 Introduction

Liposarcoma (also known as atypical lipomatous tumor^[1]^) is the most common soft-tissue tumor. It is a rare malignant tumor of embryogenic mesodermal origin presenting in the following histologic variants: well-differentiated, myxoid, round cell, pleomorphic, and dedifferentiated^[2]^).

Rarely located in the gastrointestinal tract, liposarcomas can develop in the extremities or trunk, but it is the retroperitoneum the most common location,^[3]^ as they account for approximately 15% of all retroperitoneal sarcomas.^[4]^ The incidence of retroperitoneal liposarcomas is 0.3 to 0.4 per 100,000 individuals.^[4]^ The 2 most common retroperitoneal types are well-differentiated (WDLPS) and high-grade dedifferentiated (DDLPS). These 2 variants share the molecular hallmark of MDM2 gene amplification which differentiates these from other retroperitoneal tumors.^[5]^

Retroperitoneal liposarcomas have a higher incidence in the 6th and 7th decade, they present with symptoms that vary and may be nonspecific such as bleeding, weight loss, and abdominal pain.^[6]^ Usually, retroperitoneal liposarcomas reach large sizes as the retroperitoneal region offers large for them to grow asymptomatically.^[5,7]^ We report a case of a retroperitoneal laparoscopically resected WDLPS along with a literature review of all the published retroperitoneal WDLPS cases in women.

## 2 Case report

**Figure 1:**
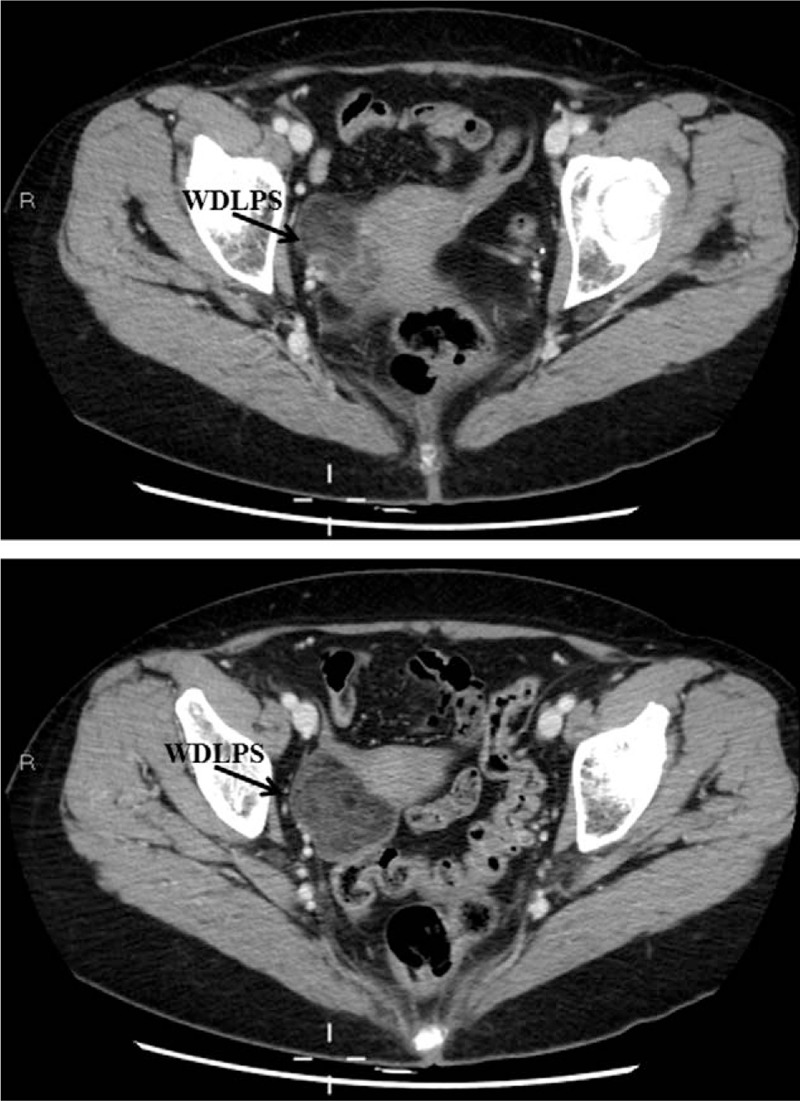
Computed tomography scan misdiagnosed a well-differentiated liposarcoma (WDLPS) as an ovarian mature teratoma because the well-capsulated homogeneous fatty components. WDLPS was located in the right pelvis close to the uterus corpus and cervix.

**Figure 2:**
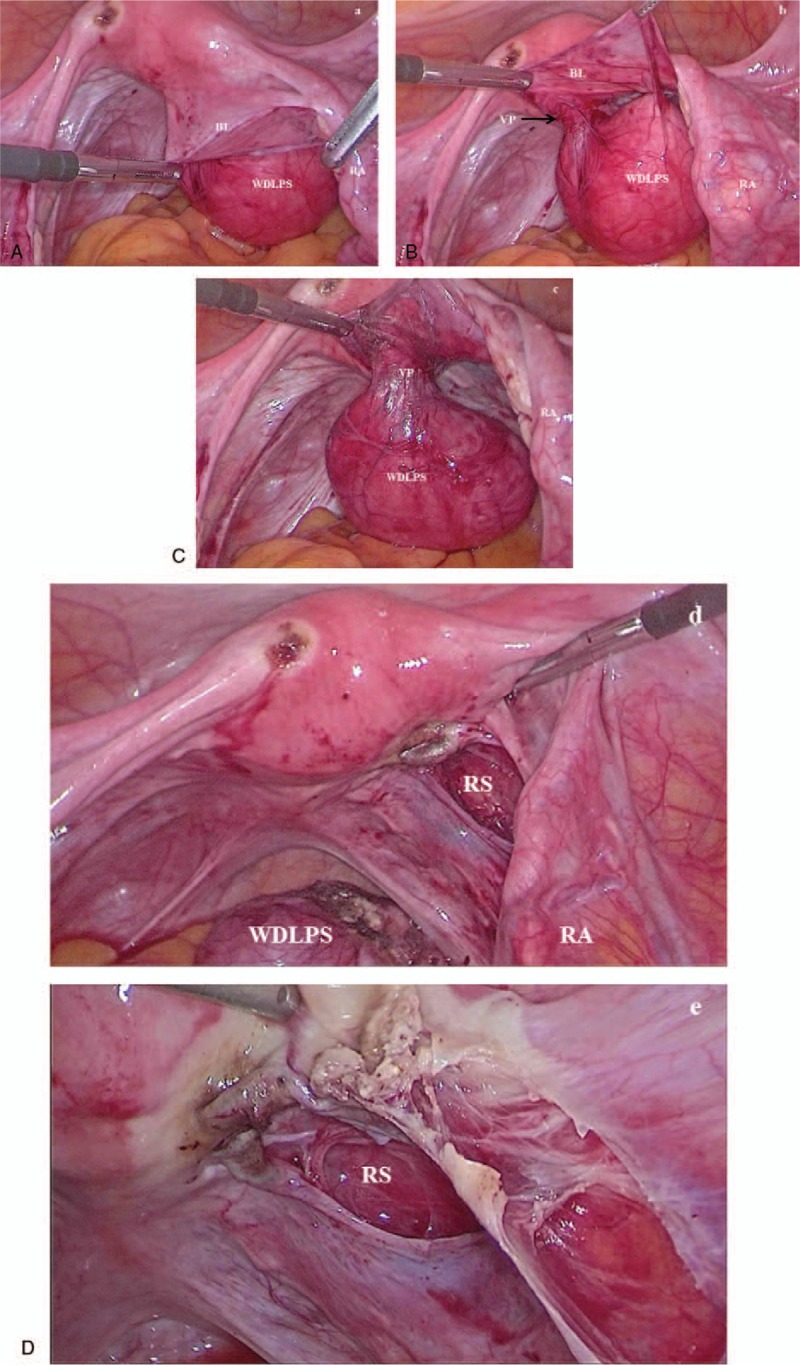
Laparoscopic radical resection: (A) a well-differentiated liposarcoma (WDLPS) was found in the posterior sheet of the right broad ligament (BL), while the right adnexa (RA) was normal; (B) during enucleation of WDLPS a vascular peduncle (VP) was found and (C) isolated; (D) WDLPS was radically resected, retroperitoneal space (RS) was free of disease; (E) a bilateral salpingoophorectomy was also performed.

**Figure 3:**
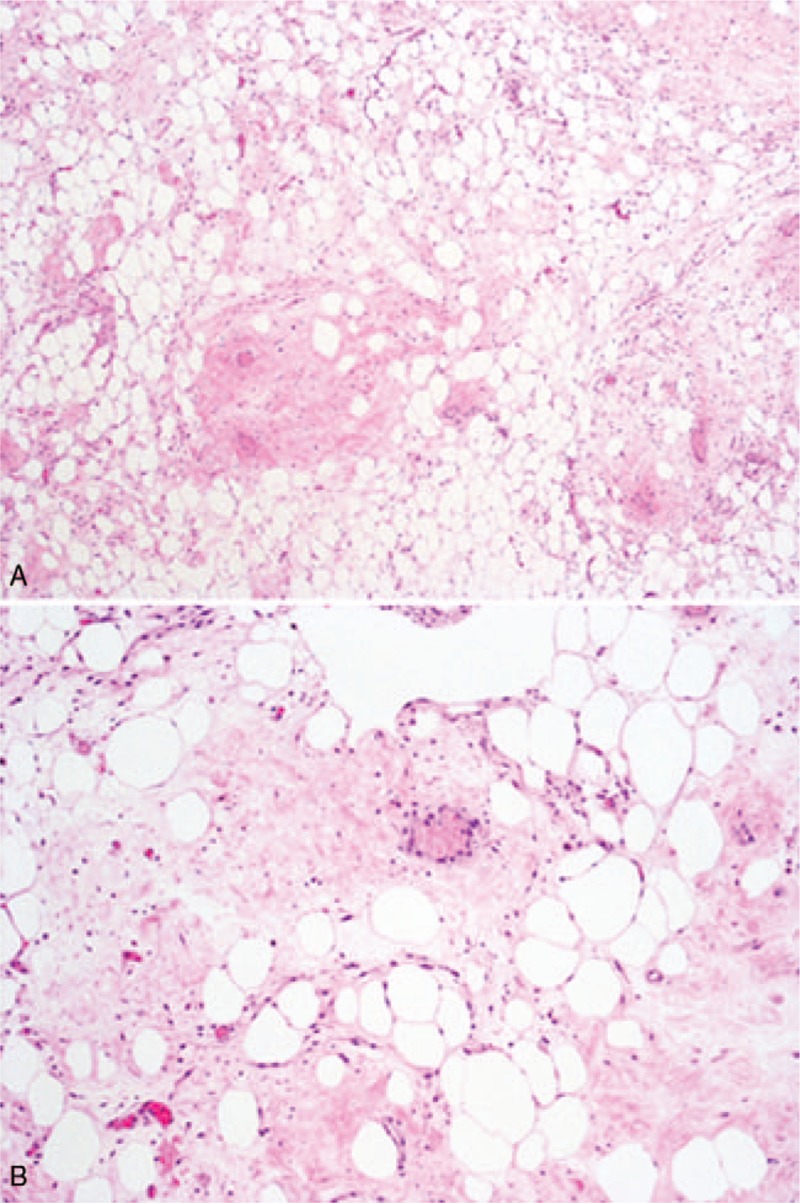
(A) Low-power view of well-differentiated liposarcoma composed entirely of a mature adipocytic proliferation, showing significant variation in cell size. (B) High-power view shows focal nuclear atypia in both adipocytes and stromal cells. The fibrous component may occasionally represent the majority of the neoplasm.

A 60-year-old woman was referred for surgery with the diagnosis of right ovarian mature teratoma. The diagnosis of ovarian teratoma was made due to a “fat fluid level” noted on transvaginal ultrasound, and confirmed on computed tomography (CT) scan (Fig. 1). The patient was asymptomatic, tumor markers were in the normal range. The adnexa and the uterus appeared to be normal at laparoscopy, a 5-cm retroperitoneal capsulated solid mass was noted in the posterior sheet of the right broad ligament (Fig. 2). The mass was radically resected and retrieved in a bag. Prophylactic bilateral salpingoophorectomy and endometrial biopsy were also performed. On hystology, adipocyte proliferation with different maturation stages was noted, as well as spindle cells with hypercromatic nuclei, inflammatory cells, and mast cells. The diagnosis of WDLPS (Fig. 3) was made. The patient's postoperative course was uneventful. Upon discharge, abdominal and pelvis CT scan as well as magnetic resonance image (MRI) were offered alternately every 6 months. Eighteen months after WDLPS resection the patient was disease free.

### 2.1 Ethical review

Ethical approval was not necessary for case report publication; and patient written informed consent was obtained to collect data and images for publication.

## 3 Review of literature

### 3.1 Systematic review of the literature

We reviewed the literature concerning on retroperitoneal WDLPS in women using PubMed until August 2018, using the following key words “well-differentiated liposarcoma,” “retroperitoneal well-differentiated liposarcoma,” “well-differentiated liposarcoma of retroperitoneum,” “atypical lipomatous tumor,” “retroperitoneal atypical lipomatous tumor,” “atypical lipomatous tumor of retroperitoneum.” A total of 36 retroperitoneal WDLPS were found; in 4 cases, few data were reported, so 32 cases were included in the review.^[2,7–35]^

### 3.2 Clinical features

Table 1   shows the main clinical features of all 32 retroperitoneal WDLPS.

**Table 1:**
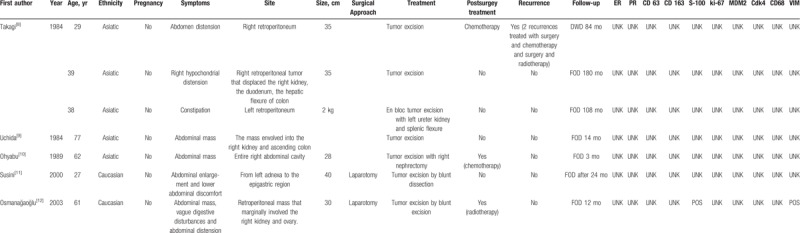
Summary of the clinical and pathologic characteristics of 31 cases of retroperitoneal well-differentiated liposarcomas reported in literature.

**Table 1 (Continued):**
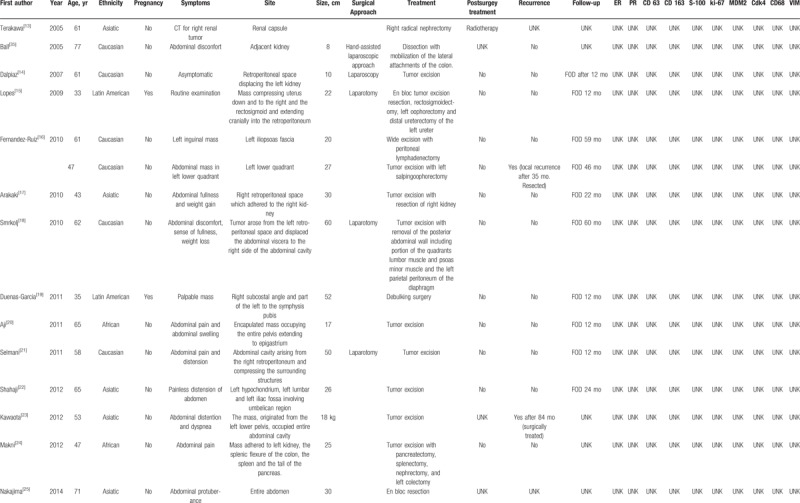
Summary of the clinical and pathologic characteristics of 31 cases of retroperitoneal well-differentiated liposarcomas reported in literature.

**Table 1 (Continued):**
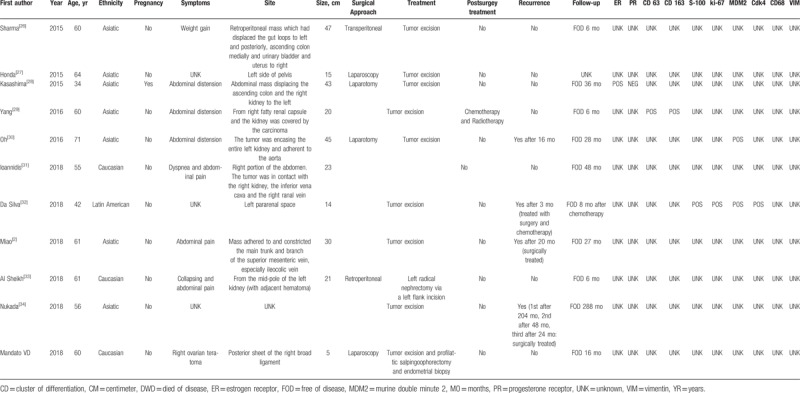
Summary of the clinical and pathologic characteristics of 31 cases of retroperitoneal well-differentiated liposarcomas reported in literature.

Mean age at presentation was 54.2 years, SD ±13.8. Three out of 31 (9.7%) patients were pregnant at the time of diagnosis.

Most of retroperitoneal WDLPS occurred in both abdomen and pelvis, only in 1 case (3.2%) WDLPS was confined in the pelvis.^[27]^ The tumor size was available in 25/32 (78.1%) patients, ranging from 8 to 60 cm, with a mean size of 29.7 cm (SD ±13.3). In 2 cases, the weight of the tumor was reported, 2 kg^[8]^ and 18 kg,^[23]^ respectively. Symptoms were reported in 29/32 (90.6%) patients, consisting in abdominal distension 10/29 (34.5%), pain 8/29 (27.6%), mass 7/29 (24.1%), dyspnea 2/29 (6.9%), weight gain 2/29 (6.9%), weight loss 1/29 (3.4%), constipation 1/29 (3.4%), syncope 1/29 (3.4%), while 2/29 (6.9%) patients were asymptomatic. The most common surgical approach was laparotomy, as minimally invasive surgery was chosen in only 3 cases (9.4%).^[14,27,35]^ WDLPS was completely excised without multiorgan resection in 23/32 (71.9%) patients,^[2,8,9,11,12,14,16,20–23,25–32,34,35]^ in 9/32 (28.1%) patients required a more aggressive surgery.^[8,10,13,15–19,24,33]^ Postsurgical treatment was reported in 28/32 (87.5%) patients: 23/28 (82.1%) patients received no adjuvant therapy, 2/28 (7.1%) patients received chemotherapy,^[8,10]^ 2/28 (7.1%) patients received radiation therapy,^[12,13]^ and 1/28 (3.6%) patient received both chemo and radiotherapy.^[29]^

### 3.3 Follow-up data

Information on recurrence was not available for 3/32 (9.4%) patients. In 22/32 (68.7%) patients, the disease did not recur, while 7/29 (24.1%) patients had 1 or more recurrences. Of note, 1/7 (14.3%) patient had 3 recurrences,^[33]^ 1/7 (14.3%) patient had 2 recurrences,^[8]^ the 1st recurrence was after 24 months and was treated by surgery and chemotherapy instead the 2nd recurrence was treated by surgery and radiotherapy, patient died of disease 84 months from the diagnosis. Notably, this patient had received adjuvant chemotherapy after 1st surgery.^[8]^ One patient of 7 (14.3%) had 1 recurrence that was treated with surgery and chemotherapy, she was FOD at 8 months from relapse.^[31]^ Four patients of 7 (57.1%) had 1 recurrence that was surgically treated, 1 patient recurred after 16 months,^[29]^ 1 after 20 months,^[2]^ 1 after 35 months,^[16]^ and 1 after 84 months.^[23]^ After surgery 3 patients remained FOD^[2,16,29]^ while we did not find follow-up information about 1 patient.^[23]^ Survival information were available for 27/32 (84.4%) patients, 26/27 (96.3%) patients were FOD and 1/27 (3.7%) patient died with disease (DWD) 84 months after diagnosis.^[8]^ Median survival (OS) was 24 months (ranging from 3 to 288 months) and the disease-free survival (DFS) was 22 months (ranging from 3 to 204 months). Among FOD patients, 16/26 (61.5%) had been treated with only tumor excision, 9/26 (34.6%) with tumor excision plus multiorgan resection and 1/26 (3.8%) patient with surgery plus adjuvant chemo and radiotherapy.^[29]^ Five of 26 (19.2%) FOD patients had 1 or more recurrences.^[2,16]^ In these patients, median DFS was 20 months, median OS was 28 months (ranging from 11 to 288 months). One of 27 (3.7%) patients died after 2 recurrences.^[8]^

## 4 Discussion

Today 32 cases of WDLPS have been reported in literature, women (mean age 54.2 years) were younger than commonly reported.^[6]^ Only 3 cases were treated laparoscopycally.^[14,27,35]^ as tumors were smaller than commonly reported. Radical tumor resection represents the gold standard for treatment.^[5]^ The 5-year survival rate for retroperitoneal WDLPS is 83%.^[2]^ WDLPS have no risk of metastasis but a high risk (60%) of local recurrence because defects of the retroperitoneal fat might create a niche for recurrence.^[5]^ In our review, the risk of recurrence was 24.1%: 7 patients had been previously treated with surgery only,^[2,8,16,23,29,31,33]^ whereas 2 patients received also chemotherapy^[8,31]^ and 1 patient also received radiotherapy at the time of 2nd recurrence.^[8]^ Because these tumors are classically chemo and radio resistant, surgical resection is essential to avoid recurrence. Usually radical surgery requires extensive multiorgan resection because the majority (80%) of retroperitoneal sarcomas are asymptomatic abdominal masses.^[5,7,28]^ In our review, the mean tumor size was of 29.7 cm (SD ±13.3 cm), ranging from 8 to 60 cm and extensive multiorgan resection was required in 28.1% of cases. Most of cases were diagnosed because presenting as huge abdominal/pelvic masses associated with aspecific symptoms such as abdominal discomfort/pain,^[2,11,17,18,21,31,35]^ constipation,^[8]^ dyspnea,^[23,31]^ weight gain,^[17,26]^ or loss.^[18]^ As in our patient, 3/28 (10.7%) cases of retroperitoneal WDLPS were accidentally diagnosed.^[13–15]^ Although in our case WDLPS was misdiagnosed with an ovarian benign teratoma, CT scan with intravenous contrast is considered the diagnostic technique of choice for WDLPS.^[5]^ In a retrospective blinded review of imaging in patients with retroperitoneal liposarcoma, an area of focal nodular/water density was found to be a specific (97.8%) marker for DDLPS and had showed a negative predictive value of 100%, suggesting that most WDLPS diagnosis can be strongly suggested on imaging alone.^[36]^ MRI is an option for patients with contrast allergies or when more precise definition of anatomy is needed.^[5]^ When preoperative imaging is not pathognomonic for WDLPS, image-guided core-needle biopsies should be obtained particularly when neoadjuvant treatment is take into consideration. The risk of tumor seeding along the biopsy track is minimal and it is not a reason to avoid such procedure.^[37]^ However, areas of potential dedifferentiation could be missed because of the large size, with a false negative rate of over 50%.^[38]^ On the contrary, open biopsy via laparotomy or laparoscopy is not recommended because of the risk of peritoneal contamination and seeding, and it may also distort tissue planes needed for subsequent resection.^[39]^ If a retroperitoneal mass is discovered incidentally during surgery performed for other reasons, an open core-needle biopsy should be considered if it can be performed without peritoneal contamination, otherwise no further action should be taken until adequate imaging is obtained. Intraoperative frozen sections have been shown to be not very useful. An operative decisions should only be made after reviewing the final pathology and imaging studies.^[40]^ In our case, we discovered incidentally WDLPS that was radically resected and sent for frozen section suggesting a tumor with adipocytes and inflammatory cells. Radical excision was obtained thank to the well-defined margins of the tumor and the scant presence of fatty tissue in the broad ligament. High degree of adipocyte differentiation makes it difficult to distinguish between WDLPS and normal retroperitoneal tissue. Laparoscopic magnification could help distinguish disease from the surrounding tissues. When the final pathology report showed WDLPS, an MRI was repeated to exclude residual disease and other localizations. Retroperitoneal WDLPS are rare and locally advanced at the time of diagnosis. Radical resection represents the only effective treatment. When feasible for tumor characteristics (lesion location, size, shape) and surgical skills, laparoscopic approach should be preferred, as it allows radical dissection of the tumor with the benefit of mini-invasive approach.^[14,27,35]^ Laparoscopy could cause less adhesions making a 2nd surgery easier in case of recurrence. However, tumor spillage should be avoided because the risk of abdominal and trocar site implant is not negligible ^[41]^ Four cases of retroperitoneal WDLPS were successfully treated by laparoscopy so far. Despite CT scan is considered the gold standard to diagnose WDLPS it is important to stress that it is possible to misdiagnose WDLPS with mature ovarian teratoma that has a different prognosis and does not require follow-up. Actually laparoscopy could represent a safe and effective technique to diagnose and treat retroperitoneal WDLPS.^[14,27,35,42]^

## Author contributions

**Conceptualization:** Vincenzo Dario Mandato, Lorenzo Aguzzoli.

**Data curation:** Valentina Mastrofilippo.

**Writing – original draft:** Vincenzo Dario Mandato, Loredana De Marco.
